# Intraoperative Neuromonitoring for Pediatric Pelvic Tumors

**DOI:** 10.3389/fped.2022.949037

**Published:** 2022-08-30

**Authors:** Alessandro Crocoli, Cristina Martucci, Franco Randi, Viviana Ponzo, Alessandro Trucchi, Maria Debora De Pasquale, Carlo Efisio Marras, Alessandro Inserra

**Affiliations:** ^1^Surgical Oncology Unit, Department of Surgery, Bambino Gesù Children’s Hospital – IRCCS, Rome, Italy; ^2^General Surgery Unit, Department of Surgery, Bambino Gesù Children’s Hospital – IRCCS, Rome, Italy; ^3^Neurosurgery Unit, Department of Neuroscience and Psychiatry Sciences, Bambino Gesù Children’s Hospital – IRCCS, Rome, Italy; ^4^Surgical Andrology Unit, Department of Surgery, Bambino Gesù Children’s Hospital – IRCCS, Rome, Italy; ^5^Hematology/Oncology Unit, Department of Pediatric Hematology/Oncology Cell and Gene Therapy, Bambino Gesù Children’s Hospital – IRCCS, Rome, Italy

**Keywords:** neuromonitoring, children, pelvic surgery, evoked potentials, cancer

## Abstract

**Background:**

Tumors of the pre-sacral and sacral spaces are a rare occurrence in children. Total tumor excision is required due to the significant risk of relapse in the event of partial surgery, but the surgical procedure may lead to postoperative problems such as urinary, sexual, and anorectal dysfunctions. Intraoperative neuromonitoring (IONM) has gained popularity in recent years as a strategy for preventing the onset of neurologic impairments by combining several neurophysiological techniques. The aim of our study is to describe the experience of Bambino Gesù Children’s Hospital in the use of IONM in pediatric pelvic surgery.

**Materials and Methods:**

The data of patients treated for pelvic malignancies at Bambino Gesù Children’s Hospital from 2015 to 2019 were retrospectively collected. All patients were assessed from a neurologic and neuro-urologic point of view at different time-points (before and immediately after surgery, after 6 months, and 1-year follow-up). They were all monitored during a surgical procedure using multimodal IONM including transcranial motor evoked potentials (TcMEP), triggered-EMG (t-EMG), pudendal somatosensory evoked potentials (PSSEP), and bulbocavernosus reflex (BCR).

**Results:**

During the study period, ten children underwent pelvic tumor removal at our Institution. In all cases, intraoperative neurophysiological recordings were stable and feasible. The preservation of neurophysiological response at the same intensity during surgical procedures correlated with no new deficits for all neurophysiological techniques.

**Discussion:**

Although the impact of the IONM on surgical strategies and clinical follow-up is unknown, this preliminary experience suggests that the appropriate use of several neurophysiological techniques can influence both the radicality of pelvic tumor removal and the neurological and urological outcome at clinical follow-up. Finally, because of the highly complex anatomy and inter-individual variances, this is especially useful in this type of surgery.

## Introduction

Tumors involving sacral and pre-sacral space are a rare condition in the pediatric population that occurs with an incidence of approximately 1 in 40,000 with a male to female ratio of 1:3–1:4 (1). The presacral space is the site of fusion of all germinal layers in the developing fetus, where a wide range of malignancies may arise; among these, sacrococcygeal teratoma (SCT) represents the most frequent benign tumor (2). Considering the high risk of relapse in case of incomplete surgery, radical surgery for uterine, rectal, and prostate tumors should be mandatory. However, it could hesitate to postoperative complications such as urinary, sexual, and anorectal dysfunctions due to intraoperative injury of pelvic splanchnic and pudendal nerves; furthermore, it has been reported that deficits of parasympathetic, sympathetic and somatic innervation of the bladder, and the urethra could occur in children after surgery for pelvic neoplasms (3). As a result, numerous efforts have been made in pelvic surgery to preserve the complex autonomic nerve distribution of this anatomic area. In the last decades, Intraoperative neuromonitoring (IONM) has become an established tool to assess neurological functions during surgeries involving the nervous system (4–8); IONM can assist the surgeon in preventing and detecting neurological problems before they become irreparable, improving the patient’s neurologic outcome. In case of surgery of pre-sacral and sacral space, continuous electromyography (c-EMG) recordings, compound muscle action potentials (cMAP) evoked from legs and sphincter muscles, and somatosensory evoked potentials (SSEPs) are advised (9, 10). Minimally invasive IONM, consisting of simultaneous electromyography (EMG) of the internal anal sphincter and manometry of the urine bladder, is commonly used during rectal cancer surgeries (7, 11–13). Furthermore, recent neurophysiological studies have demonstrated that the bulbocavernosus reflex (BCR) has prognostic significance for bladder diseases (14). The BCR has been recognized as a clinician’s tool for assessing the integrity of the S2-S4 reflex arc and, as a result, detecting neurogenic “sacral dysfunction” such as lower urinary tract and erectile dysfunction fast (15).

Here we present a retrospective study about the use of IONM in children undergoing pelvic surgery, assessing its feasibility in this specific population. The second aim of our study is to assess whether BCR abnormality could be predictive of neurogenic lesions in the pediatric population and therefore be used to influence surgical decisions.

## Materials and Methods

We retrospectively collected data from anesthesia records and electronic charts of children treated for pelvic malignancies from 2015 to 2019 at Bambino Gesù Children’s Hospital. All data including demography, clinical signs and symptoms, management, postoperative complications, histology, and recurrence were obtained for each patient.

All patients were clinically assessed during admission (t0), immediately after discharge (t1), at 6 months after surgery (t2), and at 1-year (t3) follow-up. The urodynamic evaluations were performed immediately before and after surgery.

Ethical review and approval was not required for the study on human participants in accordance with the local legislation and institutional requirements, as well as written informed consent from the patients or participants’ legal guardian.

### Anesthesia Protocol

Different studies have previously described how inhaled anesthesia (IA) and total intravenous anesthesia (TIVA) may influence differently the quality of IONM recordings (16); following their instructions, general anesthesia was induced with bolus and maintained by Propofol 2% (Cpt 3,0 mcg/ml) and remifentanil (0.25–0.5 mcg/kg). Rocuronium bolus (0.6–1.0 mg/kg) was administered to ease orotracheal intubation. No further dose of muscle relaxant was administered during the surgery. Bispectral index (BIS) values were used to monitor the depth of anesthesia (17, 18). All patients were monitored using arterial blood pressure electrocardiography activity and level of O_2_ saturation.

### Neurophysiological Assessment

The neurophysiological setup was tailored depending on different tumor sites. A product named 32-channel Cadwell Cascade Pro from Cadwell Industries Inc., Kennewick, WA, United States was used to map relevant neural structures and monitor their functional integrity continuously during the surgery. The most part of the patients was monitored using a multimodal neurophysiological approach including transcranial motor evoked potentials (TcMEP) from the upper (abductor pollicis brevis performing as muscle control) and lower extremities [tibialis anterior (TA), adductor hallucis, and external anal sphincter (EAS)], SSEPs, continuous electromyography (c-EMG), and BCR. Each recording was carried out immediately before the surgical incision (baseline) and approximately every 15 min from the beginning until the end of surgery. Triggered-EMG (t-EMG) was available and performed by using electrical stimulation as required by the surgeon. For all tests, impedance was maintained below 5 K-Ohms.

### Transcranial Motor Evoked Potentials

Transcranial Motor Evoked Potentials (TcMEP) stimulation was delivered by transcranial electrical stimulation *via* corkscrew electrodes in the scalp at C1/C2 for monitoring lower extremities and C3/C4 for upper extremities. A train of 5–7 pulses was set at 50–75 μs of duration and stimulation intensity was maintained at the supramaximal level in a range of 120–380 volts.

### Somatosensory Evoked Potentials

For SSEP, lower extremities were stimulated using twisted paired needles positioned over the tibial nerve at the ankle. SSEP signals were recorded using corkscrew electrodes inserted over the scalp at Cz-2 (active electrode) and Fz (reference electrode), according to the International 10–20 EEG positioning and the somatotopic representation of the primary somatosensory cortex (10). For each average, 200 traces were performed to produce a well-defined EP tracing. Time-base was set at 10 ms/div. Notch filters were set at 50 Hz. In addition, to test pudendal somatosensory evoked potential (PSSEP), a surface electrode (Cadwell Industries, Inc., Kennewick, WA, United States) was fixed to the glans of the penis or clitoris and corkscrew electrodes were set over Cz (central point at the sagittal midline from nasion to inion) and Fz (frontal point) as reference electrode (19). The current intensity was set up to allow twitch muscle contraction.

### Electromyography Activity

Lower-extremity EMG activity was monitored using 13-mm twisted paired needle electrodes subdermal (Cascade Kennewick, WA) from tibialis anterior (TA) as the control recording and external anal sphincter (EAS). The EAS electrodes were placed over the anus when the patient was already positioned prone on the operating table. The electrodes were secured using adhesive tape while a rolled sponge was placed to guarantee more space between the electrodes. We performed both free-running and triggered EMG to identify, respectively, surgical manipulation (cold irrigation, traction, and coagulation) and mapping nerve root or rootlets. Bandpass filters were set at 10–5000 Hz and time-base at 200 ms for division. Electrical stimulation was performed using by bipolar stimulator probe. Pulse duration was set between 75 and 200 μs while current intensity can be ranged from 0.1 to 10 mA. Any significant intraoperative spontaneous and triggered EMG activity was reported to the surgeon.

For monitoring the external urethral sphincter (EUS), a custom-made twisted disposable subdermal electrode (Cascade Kennewick, WA) was secured around a Foley catheter using adhesive tape, respectively, 2–3 cm below the balloon for females and 2–5 cm for males. A ground electrode was positioned over the leg region.

### Bulbocavernosus Reflex

Two bipolar needle electrodes were inserted on both sides of the external anal sphincter (20, 21) and were adequately secured using adhesive tape in order to prevent their displacement and outdistanced by rolled sponge. Surface EMG electrodes were used to perform pudendal stimulation. The cathode was positioned on the proximal penis or clitoris while the anode on the distal penis or labia majora, respectively, for males and females. Just oligosynaptic reflex may be recorded intraoperatively because of the high susceptibility to general anesthesia (mainly for volatile agents) of BCR. The stimulating rate of 2.3 Hz and 20–40 mA of current intensity were considerate optimal parameters. No averaging was used.

## Results

Ten children who underwent pelvic tumor removal under general anesthesia between January 2015 and May 2019 at Bambino Gesù Children’s Hospital were monitored intraoperatively for a high risk of postoperative urinary and bowel dysfunction. Four of them were male; the median age was 3.9 years (range 4–8 years).

All demographic and clinical data are summarized in Table 1; neurophysiological features are reported in Table 2. In all cases, radical resection was performed.

**TABLE 1:** Demographic and clinical data.

Age (y)	Sex	Urodynamic testing (t0)	Urodynamic testing (t1)	Clinical exam (t0)	Clinical exam (t1)	Clinical exam (t2)	Clinical exam (t3)	Chemotherapy	Histology
4	F	Normal	Normal	Fecal incontinence, spontaneous diuresis, abdominal pain	Unchanged	No urinary and fecal retention	No urinary and fecal retention	Received	Endodermal sinus tumor (EST)
2	M	NA	NA	Paraparesis	Unchanged	NA	NA	Received	Mature teratoma
10	M	NA	NA	Normal	Unchanged	Unchanged	Unchanged	Received	Embryonic Rhabdomyosarcoma
1	F	Neurogenic detrusor overactivity	Neurogenic detrusor overactivity	No other neurological deficit	Unchanged	Unchanged	Unchanged	Received	Endodermal sinus tumor (EST)
1	F	NA	NA	Constipation for long time	No sphincter deficit	Unchanged	Unchanged	Not received	Mature teratoma
1	F	NA	NA	Opsoclonus, myoclonus, postural instability	Unchanged	Unchanged	Unchanged	Not received	Presacral neuroblastoma
8	F	NA	NA	Normal	Unchanged	Unchanged	Unchanged	Not received	Abdominal ganglioneuroma
3	M	NA	NA	Normal	Unchanged	Unchanged	Unchanged	Received	Embryonic rhabdomyosarcoma
1	M	NA	NA	Normal	Unchanged	Unchanged	Unchanged	Received	Pelvic neuroblastoma
8	F	NA	NA	Caudal regression syndrome, neurogenic bladder, anorectal malformation	Unchanged	Unchanged	Unchanged	Not received	Median raphe cyst

**TABLE 2:** Neurophsiological findings: assessment of significant alteration for several intraoperative neuromonitoring (IONM) techniques.

Patient	EMG	BCR	Pudendal SEP	TcMEP	IONM finding
1	No	No	No	Yes (Threshold increase)	BCR poorly reproducible from baseline
2	No	No	No	No	Threshold increase of MEP and BCR from baseline
3	No	No	Not performed	Not performed	Not performed
4	Yes	Yes	Not performed	Not performed	Neurotonic discharge of right external anal sphincter; BCR poorly reproducible elicited by right external anal sphincter in comparison from the baseline
5	No	No	Not performed	Not performed	–
6	No	No	Not performed	Not performed	–
7	No	No	No	No	–
8	No	No	No	Not performed	–
9	No	No	No	Not performed	–
10	No	No	Not performed	Not performed	Absent BCR bilaterally from baseline recordings; spontaneous activations of bilateral external anal sphincter

We used the absence of evoked potentials, decreased amplitude, or latency lengthening as warning criteria based on earlier evidence of IONM’s prognostic efficacy in the spine and rectal procedures, as previously described in literature (10, 22, 23). The maintenance of responses showed that the sensorimotor pathways are functionally intact, whereas their removal or severe amplitude deterioration may indicate damage to these tracts or their posterior roots.

Lower-limb TcMEP was performed on three patients who had neurological impairment at baseline and a higher motor threshold during presurgical evaluation; TcMEP was thus useful in providing quick information about the functional integrity of monitored muscles. We also observed that maintaining MEPs response at the same intensity as the baseline signal recorded at the end of the surgical procedure was associated with the absence of any new motor deficits.

Bulbocavernosus reflex (BCR), pudendal SEPs, and external anal sphincter EMG were performed in all patients that showed a clear radiological bladder and/or rectum tumor involvement. Clinically, four patients with germ cell tumors were affected by cauda equine syndrome, and two patients with neuroblastic tumors were reported with opsoclonus-myoclonus syndrome and low back, respectively. No clinical signs and symptoms were found in the other four patients affected by bladder embryonal rhabdomyosarcoma (n. 2), neuroblastic tumor (n. 1), and median raphe cyst (n. 1). The surgery was interrupted when BCR was lost intraoperatively, and the surgical procedure and anatomical field were assessed.

Only two children had bladder urgency and frequency prior to surgery. They found an increase in BCR threshold compared to normative values at baseline, as well as neurotonic EMG activity of anal sphincters with high-frequency, regular, and persistent signals. In all cases, the patient’s frequency and urgency improved immediately after surgery, and bladder function returned to normal within 2 weeks of the tumor removal.

We were able to successfully record t-EMG responses from the external urethral sphincter muscles in all of the patients. The morphology of EUS’s t-EMG responses was consistent and repeatable. Moreover, in two patients we successfully managed to evoke TcMEP from EUS.

No technical complications were reported for neurophysiological tests.

No patients suffered from clinical or radiological deterioration in the follow-up (t1, t2, t3). No significant differences were found in postoperative urodynamic assessment in comparison to preoperative.

## Discussion

Because the pelvic splanchnic nerves, the hypogastric nerve, and the pelvic nerve plexus may be involved, pelvic surgery has the potential to be harmful, as already reported in literature (3).

To our knowledge, this is the first study exploring the use of IONM in pediatric pelvic surgery. Poor nerve visibility may indicate a significant risk of nerve damage with eventual anorectal and/or urogenital dysfunction due to a lack of neuroanatomical expertise and other patient-related variables (pelvis anatomy, past surgical procedures, etc.). Large multicenter studies on rectal cancer treatment have revealed that urogenital and sexual dysfunctions are still a prevalent postoperative consequence that can be induced even by a unilateral autonomic nerve lesion alone (4).

Our findings indicate that using a customized IONM to carefully preserve nerves during pelvic surgery may help to improve or maintain a stable neurological outcome. Intraoperative neurophysiological recordings are stable and possible for all patients, according to our experience.

Intraoperative neuromonitoring (IONM) has grown in popularity over the last three decades as a beneficial tool for protecting patients from neurological injury during surgical procedures in both adults (24–26) and children (17, 18, 27). The practice of IONM, well established in neurosurgical procedures involving supratentorial, infratentorial, and spinal structures (24, 26, 28), is nowadays spreading in other surgical fields as well as general surgery. However, currently, no specific data about the use of IONM in pediatric pelvic tumor excision are found.

Although the impact of the IONM on surgical strategies and clinical follow-up is still unknown, our preliminary experience suggests that the IONM’s relevance may influence both the radicality of surgical therapy and the neuro-urological outcome. Furthermore, IONM has sometimes allowed modifying surgical plans, making them more aggressive while yet remaining safe, even in tumors with difficult localization. As suggested by Krassioukov et al. (8), we shared the decision to perform both SSEP and EMG of lower-limb muscles, EAS, and EUS. These tests are considered essential for producing optimal electrophysiological feedback for surgical decision-making. Because of the high specificity and sensitivity in diagnosing neurological impairments, we chose to utilize IONM with TcMEP elicited from the same muscle as EMG, contrary to what has already been described in the literature (8).

Aside from neurologic impairment, one of the most serious risks is intraoperative bladder dysfunction caused by the tumor’s pelvic involvement. As reported in recent studies (29), bladder dysfunction caused by a lack of urological surveillance accounts for roughly half of all bladder dysfunction in the last decade. According to another survey (30), one out of every three children who underwent resection of SCT during childhood had persistent issues in adult age. As a result, we decided to broaden our neurophysiological evaluation to include the BCR test. As is well known, BCR is an important technique for predicting and preserving postoperative sacral function (8). When the BCR response is preserved at the end of the surgical treatment, the postoperative outcome is maintained in over 90% of cases after 6 months (14, 31, 32). Traditionally, BCR allows to test both the lower tract urinary and anorectal function, as well as to determine sphincter activity in connection to micturition or feces, using EAS EMG. Many papers have investigated the clinical significance of BCR in predictive postoperative voiding function (14). BCR, according to these authors, could be used to assess the anatomical and functional integrity of the sensorimotor reflex arc, which includes both parasympathetic and pudendal innervation (33, 34) and thus show how intraoperative BCR changes could be used to predict bladder function.

The autonomic nervous system (pelvic parasympathetic) and the somatic nervous system (pudendal nerve) are both governed by very similar but distinct pathways (35). Inter-neuronal networks promote the integration of these systems. No somatic or parasympathetic cord lesion should be indicated by an intact BCR and/or EAS transcranial MEP (14, 36). Some authors described a revised IONM approach that included both EMG of the internal anal sphincter and cystomanometry of the urine bladder in order to precisely evaluate bladder function (4, 7). In addition or alternatively, some authors had proposed, during spine surgery, the use of a custom-made urethral ring electrode in adult patients to get t-EMG (37) and analyze the reaction of the external urethral sphincter muscle to repetitive transcranial electrical stimulation for spine surgery (rTES) (22). Because of the highly complicated anatomy and inter-individual variability in both adults and children, pelvic surgery might undoubtedly benefit from the use of these types of electrodes (33, 38, 39). In this regard, neural structures, notably the pelvic splanchnic nerves, are difficult to identify macroscopically due to their intricate network of fibers.

In this study, we reported our results from two pediatric cases in which we monitored the urethral sphincter selectively. Despite the fact that it was more difficult to utilize this type of device in children due to the smaller size of the Foley catheter and underdeveloped myelination of the motor pathways (40), both EMG and TcMEP induced by EUS were steady and practical. Furthermore, we encountered no technological challenges in modifying and positioning electrodes. During the awakening process, the patient did not express any overall discomfort.

In conclusion, our experience has demonstrated the utility of IONM in complex fields such as pediatric pelvic surgery requiring a radical resection and a satisfactory neurological/urological outcome in the clinical follow-up. IONM could be effective for both identifying potential problems before they become permanent and immediately influencing surgical strategy. In this way, IONM aids in the protection and preservation of relevant neurological structures during surgeries, as well as the identification and selection of structures for resection while sparing other structures. As a result, pediatric IONM might benefit from the routine use of specialized devices that, nowadays, are frequently not present or available for children.

## Data Availability Statement

The raw data supporting the conclusions of this article will be made available by the authors, without undue reservation.

## Author Contributions

CM, AC, and FR conceived the study and wrote the first draft of the manuscript. AI worked on the drafts and re-edits of the manuscript. All the authors contributed to study design, analysis, data collection and interpretation of the results, reviewed the manuscript, and agreed with the decision to submit for publication.

## Conflict of Interest

The authors declare that the research was conducted in the absence of any commercial or financial relationships that could be construed as a potential conflict of interest.

## Publisher’s Note

All claims expressed in this article are solely those of the authors and do not necessarily represent those of their affiliated organizations, or those of the publisher, the editors and the reviewers. Any product that may be evaluated in this article, or claim that may be made by its manufacturer, is not guaranteed or endorsed by the publisher.
